# Evaluating Genetic Diversity and Regional Variation in Tswana Goats of Botswana

**DOI:** 10.3390/genes16060678

**Published:** 2025-05-30

**Authors:** Amantle Bonolo Chalebgwa, Phetogo Ineeleng Monau, Kethusegile Raphaka, Khanyisile Hadebe, Patrick Kgwatalala, Shalaulani James Nsoso

**Affiliations:** 1 Department of Animal Sciences, Faculty of Animal and Veterinary Sciences, Botswana University of Agriculture and Natural Resources, Private Bag 0027, Gaborone 00267, Botswana; amantlebonolochalebgwa@gmail.com (A.B.C.); pkgwatal@buan.ac.bw (P.K.); sjnsoso@buan.ac.bw (S.J.N.); 2 Center for Bio-Economy, Botswana University of Agriculture and Natural Resources, Private Bag 0027, Gaborone 00267, Botswana; 3 National Agricultural Research and Development Institute, Private Bag 00177, Gaborone 00267, Botswana; kethusegile@nardi.org.bw; 4 Agricultural Research Council, Biotechnology Platform, Private Bag X5, Ondersterpoort 0110, South Africa; mdladlak@arc.agric.za

**Keywords:** indigenous goat, animal population genetics, polymorphisms and genome annotation

## Abstract

Background/Objectives: The Tswana goat, an indigenous Botswana breed, remains genetically understudied despite its adaptation to local conditions. This study characterized its genetic diversity across regions, using Boer goats as a reference, to assess population structure, heterozygosity, and breeding patterns. Methods: Genomic DNA from Tswana goats (Southern, Central, Northwest, and research ranch populations) and Boer goats was genotyped using the Illumina Goat_IGGC_65K_v2 BeadChip. Data were analyzed in PLINK v1.9 and R v4.3.2 to compute genetic diversity indices. Results: Tswana goats showed higher genetic diversity than Boer goats, with greater minor allele frequency (MAF: 0.313 ± 0.127 vs. 0.287 ± 0.136) and expected and observed heterozygosity (*Ho*: 0.395 ± 0.019 vs. 0.367 ± 0.022, and *He*: 0.400 vs. 0.375). Regional variation emerged across the Central (*Ho* = 0.394, *He* = 0.401, and MAF = 0.320), Southern (*Ho* = 0.397, *He* = 0.399, and MAF = 0.318), Northwest (*Ho* = 0.364, *He* = 0.358, and MAF = 0.289), and research ranch populations (*Ho* = 0.394, *He* = 0.380, and MAF = 0.300). Inbreeding coefficients (F_IS_) ranged from mild inbreeding (Central: 0.019) to heterozygote excess (research ranch: −0.038), reflecting managed breeding. Conclusion: Tswana goats have high genetic diversity, with regional variation linked to breeding practices. Although regional structure suggests genetic differentiation, no distinct ecotypes were identified. These findings emphasize the need for controlled breeding to preserve genetic diversity for the Tswana goat.

## 1. Introduction

Goats thrive in diverse environments, making them one of the most prolific domesticates, with far-reaching implications for agricultural economies worldwide [1]. Botswana, with a traditional sector goat population exceeding 1.4 million, boasts a diverse landscape shaped by 4 main agroecological regions, each characterized by distinct climatic and soil conditions [2]. The Central region is classified as hardveld dominated by *Acacia tortilis* and *Colosphospermun mopane* [3]). The Southern region features a hardveld rich in *Acacia erioloba*, *Peltophorum africanum*, and *Boscia albitrunca* [3]. The Ghanzi agroecological region, predominantly covered by the Kalahari Desert, is characterized by various Acacia species [3]. In contrast, the Northwest region encompasses the Okavango Delta, a dynamic wetland ecosystem, and is classified as sandveld, with expansive grasslands and woodlands [4].

The native Tswana goat is well adapted to this varied microclimate, renowned for its resilience in the face of resource scarcity [5]. This breed plays a crucial role in the agricultural economy of Botswana, serving as a vital source of income and livelihood for local communities [6,7]. However, goats are primarily managed in communal grazing areas where fencing is prohibited, and most smallholder farmers invest little in additional feed, management efforts, or health care practices [6]. Additionally, indiscriminate mating practices without clear breeding objectives are common [7]. Despite their economic and cultural significance, limited research has been conducted to characterize the genetic architecture of Tswana goats across different agroecological regions. Given the ecological variability across Botswana, it remains unclear whether Tswana goats exhibit distinct genetic profiles shaped by their respective environments.

A previous study performed on Tswana goat genetic diversity was based on a single region and does not provide a clear picture for the whole country [7]. Another study on phenotypic characterization elucidated possibilities of different strains of Tswana goats in different regions [8]. The current study aims to address this knowledge gap by assessing the genetic diversity of Tswana goats across Botswana’s agroecological regions. By investigating whether genetic differentiation exists among goats from different regions, the research seeks to provide insights into potential ecotypic variation, inform sustainable management strategies, and support conservation efforts. These findings will contribute to the long-term viability and productivity of the Tswana goat, reinforcing its role as a key genetic resource in Botswana’s livestock sector.

## 2. Materials and Methods

### 2.1. Study Area and Sample Collection

**Figure 1:**
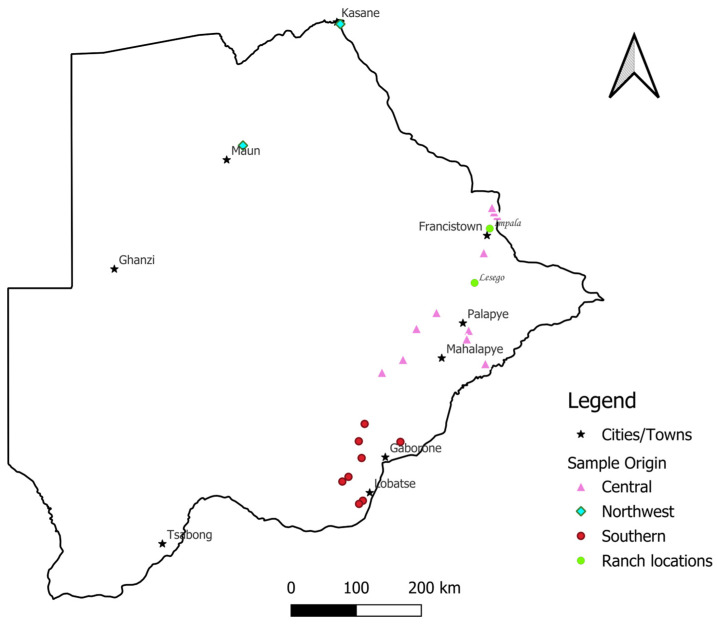
A map of Botswana showing the sample locations according to agroecological region and select major cities and towns.

A total of 192 Tswana goat hair samples from three different agro-ecological regions of Botswana were collected (Figure 1). The 3 agro-ecological regions from which the animals were sampled are the Northwest (*n* = 13) (sand-veld), Southern (*n* = 32) (hardveld), and Central (*n* = 39) regions (hardveld). No samples were collected from the Ghanzi region, as no goats identified as Tswana were found. Hair samples with intact follicles from 84 Tswana goats were collected from communal farms in these regions and stored individually in labelled envelopes. About 50–100 hairs were collected from each animal by plucking from the tail end with a pair of pliers. Villages/towns per region were randomly picked, and no more than 3 animals were sampled from the 4/5 randomly chosen farms in each area. To avoid sampling related animals, farmers were consulted for pedigree information. Regarding animal management, the animals were kept extensively and kraaled at night with minimal input, and water was occasionally provided.

The other 108 hair samples were collected from Tswana goats kept at the Impala and Lesego research ranches managed by the National Agricultural Research and Development Institute (NARDI) (Figure 1). Hair samples were collected as previously described and kept in separate labelled envelopes. The research stations are characterized under the Central region, and species such as *Colophospermum mopane* and *Vachellia erioloba* are common. The animals were also kept extensively and kraaled at night, with water provided *ad libitum* and breeding controlled [9]. To synchronize births with autumn, males are separated from females, and mating is permitted only during October and November. Other management practices include the provision of supplements; deworming; and dipping [9].

A reference population of 112 Boer goats was included in this study to provide a comparative framework for assessing genetic diversity in Tswana goats. The Boer goat genotyped data were obtained from stud breeders and commercial farms in South Africa.

### 2.2. Genotyping and Quality Control (QC)

The 192 Tswana goat hair samples were sent to France, Labogena DNA platform (Domaine de Vilvert, CS 80009, 78353 JouyenJosascedex), for genotyping with the Illumina Goat_IGGC_65K_v2 BeadChip containing 59,727 SNPs. The samples from the communal areas were assigned family IDs according to the various regions of origin such as TswanaNW (Northwest), TswanaCN (Central), and TswanaSO (Southern). The animals kept at the Lesego and Impala research ranches for research purposes were assigned the ID TswanaRSC, and the whole dataset was quality-controlled on PLINK, employing the thresholds (*--geno* 0.05, *--mind* 0.05) to ensure high data integrity. Variants on sex chromosomes and those that were on the same position were also removed. In cases where multiple variants were located at the same genomic position, only one variant was retained.

The Plink functions, *–hardy*, *--freq*, and *--het*, were used to compute the heterozygosity (observed and expected), minor allele frequencies, and inbreeding coefficient (F_IS_). The linkage disequilibrium (LD) analysis was performed using PLINK v1.9 [10] with parameters *--r2 --ld-window-kb 2000 --ld-window-r2 0.* SNP marker pairs were categorized into intermarker distance bins of 0–10, 10–20, 20–40, 40–60, 60–100, 100–200, 200–500, 500–1000, and 1000–2000 kb for further LD analysis. R v.4.3.2 [11] was employed for computing averages and standard deviations with the R package tidyverse. LD visualizations were created with the R package *ggplot2*. The effective population size (*Ne*) of the Tswana and Boer goat populations was estimated using SNeP v1.1 [12], with the following key parameters: a minimum inter-SNP distance of 10 kb, a maximum of 1 Mb, a bin width of 50 kb, 30 bins, a recombination rate of 1 × 10^−8^, and a minor allele frequency (MAF) threshold of 0.05. The plot was visualized with the R package *ggplot2*.

Principal component analysis (PCA), which involved computing genetic distances between individuals from Tswana and Boer goat populations, was employed using PLINK v.1.9 [10]. Subsequently, the generated distance matrix was imported into R v.4.3.2 software [11] for further analysis. The *cmdscale* function was applied to conduct PCA, facilitating the extraction of eigenvalues and eigenvectors. These eigenvalues, indicative of the proportion of variation captured by each principal component, were then calculated and visually represented using the *ggplot2* package within the *tidyverse* framework on R v.4.3.2 software [11].

A neighbor-joining (NJ) tree was constructed using a SNP-based distance matrix from PLINK to assess genetic relationships. The matrix was imported into R v.4.3.2 software, and the NJ tree was generated using the *ape* package and saved in Newick format for visualization on the Interactive tree of life (iTOL) [13].

## 3. Results

Three animals (1 Boer goat and 2 Tswana goats) were removed during application of quality control thresholds, and the resulting 301 animals were retained for further analysis (Table 1). The number of rare alleles (MAF < 5%) and variants with less than a 95% SNP call rate, which were excluded from analysis, were higher in the Boer goat population than the Tswana goat. The substantial removal of 62,618 SNPs based on the call rate threshold strongly suggests the presence of genotyping errors within the Boer goat data, which necessitates stringent quality control measures. A total of 10,298 SNPs were removed in the Tswana, and a further 76 variants were removed for HWE deviation (*p* < 0.00001), compared to 92 in Boer goats (Table 1).

The Tswana goat genetic diversity indices are delineated as per agroecological region in Table 2. The research ranch population (TswanaRSC) is also differentiated for the appreciation of any variation. The Central and Southern regions present the highest levels of MAF (0.320 ± 0.122 and 0.318 ± 0.123) with low inbreeding values (0.005 ± 0.038 and 0.019 ± 0.051). The high standard errors may be a result of the small sample sizes. Goats from the Northwest display reduced heterozygosity (*Ho* = 0.364 ± 0.000 and *He* = 0.358 ± 0.000) with low MAF values (0.289 ± 0.132). The research ranch animals show an excess of heterozygotes (*Ho* = 0.394 ± 0.000 and *He* = 0.38 ± 0.000) and low values for the inbreeding coefficient (−0.038 ± 0.042) with a slightly lower value for the MAF (0.30 ± 0.132), in contrast with the Southern and Central goats.

Table 3 contrasts the observed (*Ho*) and expected (*He*) heterozygosities, minor allele frequencies (MAF), and the inbreeding coefficient (F_IS_) of these populations. The Tswana shows higher *Ho* = 0.393 ± 0.121, *He* = 0.398 ± 0.112, and MAF = 0.313 ± 0.127 compared to the Boer *Ho* = 0.364 ± 0.138, *He* = 0.372 ± 0.133, and MAF = 0.287 ± 0.136, suggesting greater genetic diversity. The level of inbreeding for the Tswana goat (0.0129 ± 0.047) and the Boer goat (0.0199 ± 0.059) suggest the same contrast in these populations (Table 3).

### 3.1. Linkage Disequilibrium

**Figure 2:**
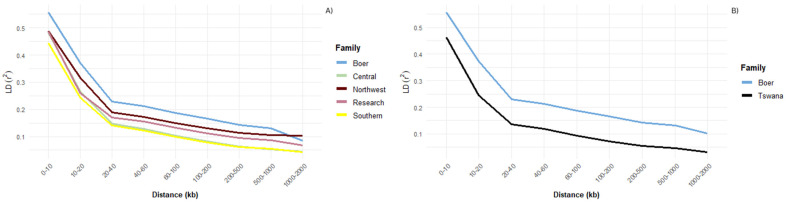
Linkage disequilibrium (LD) decay patterns with increasing marker distances. (**A**) LD decay across different agroecological regions in Tswana goats compared to the Boer goat. (**B**) Aggregate LD decay across all Tswana goats contrasted with the Boer goat.

Linkage disequilibrium (LD) decay analysis revealed distinct patterns across the goat populations. The Tswana goat populations, particularly those from the Central and Southern regions, showed notably lower average LD values, across most chromosomes, with average r^2^ values below 0.10 on chromosomes 1, 5, 9, 10, and 23, reflecting faster LD decay and higher historical recombination or greater genetic diversity (Figure 2A). Notably, chromosomes 6, 7, 12, and 25 showed the highest LD in Boer goats (r^2^ ≥ 0.18), suggesting potential regions of selection or reduced recombination (Table S1). The Northwest and research populations demonstrated intermediate LD levels (≈0.11–0.14), with peak r^2^ values reaching 0.13–0.14 on chromosomes 6, 12, and 17 (Table S1). Generally, Boer goats consistently exhibited the highest average LD values across all chromosomes when contrasted with the Tswana population, indicating extended LD and suggesting a history of more intensive selection or smaller effective population size (Figure 2B).This points to the genetic distinctiveness of the Boer population relative to the Tswana populations and suggest regional differences in selection pressure or breeding history among the indigenous Tswana goats.

### 3.2. Effective Population Size

**Figure 3:**
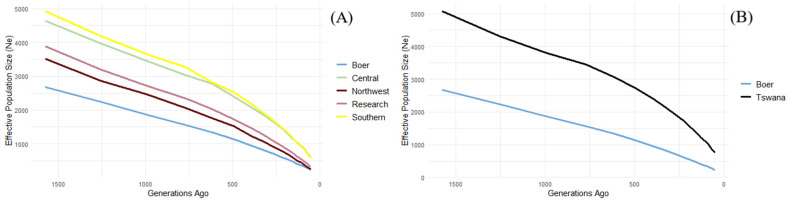
Effective population size (*Ne*) of (**A**) Tswana populations from various agroecological regions and (**B**) Tswana and Boer goats from 1576 to 53 generations ago.

Historical effective population size (*Ne*) was estimated using linkage disequilibrium across the five goat populations (Figure 3A). At 53 generations ago, *Ne* ranged from 240 in the Boer population, followed by the Northwest (243), research (319), Southern (577), and Central (593) populations. By 266 generations ago, the Southern population maintained the highest *Ne* (1667), followed closely by Central (1637), while the Boer remained the lowest at 703. At 1576 generations ago, *Ne* increased across all populations, with the Southern still leading at 4924 while the Boer remained the smallest at 2674. These differences suggest contrasting demographic histories, with the Southern and Central populations likely having experienced less genetic drift compared to the more isolated or intensively managed Boer and research groups. The general trend is that the Tswana population consistently had larger *Ne* values compared to the Boer across all time points (Figure 3B).

### 3.3. Population Structure Analysis

**Figure 4:**
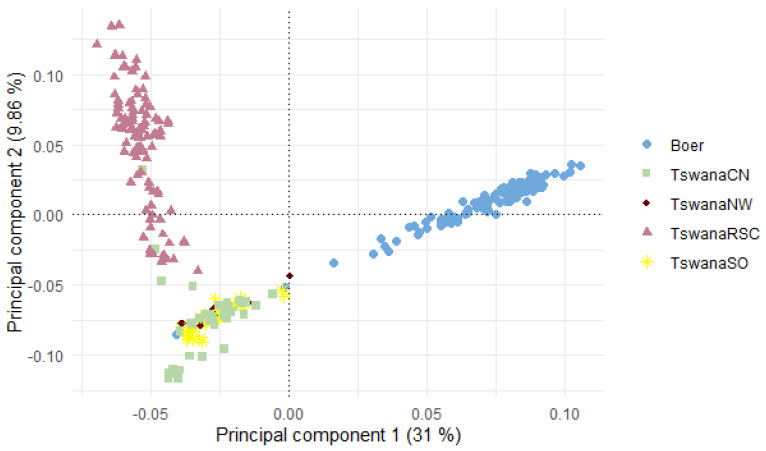
Principal component analysis (PCA) of the Tswana goat from various agroecological regions and research ranches, and that of the Boer goat.

Figure 4 shows insights into the genetic relationships between the Tswana and Boer goat populations. The first two principal components collectively account for approximately 40.86% of the genetic diversity, specifically, the first principal component (PC1) explains 31% of the overall genetic variation, whilst PC2 contributes 9.86% to the explained variability. The Boer goat population is clearly defined from the Tswana population, with three outliers inter-mingled within the Tswana goat samples. The communal populations from the Central (TswanaCN) and Southern (TswanaSO) regions cluster closely, while the Northwest (TswanaNW) samples form the majority of the outliers. The research ranch animals (TswanaRSC) appear dispersed but also show considerable differentiation from the communal cluster.

**Figure 5:**
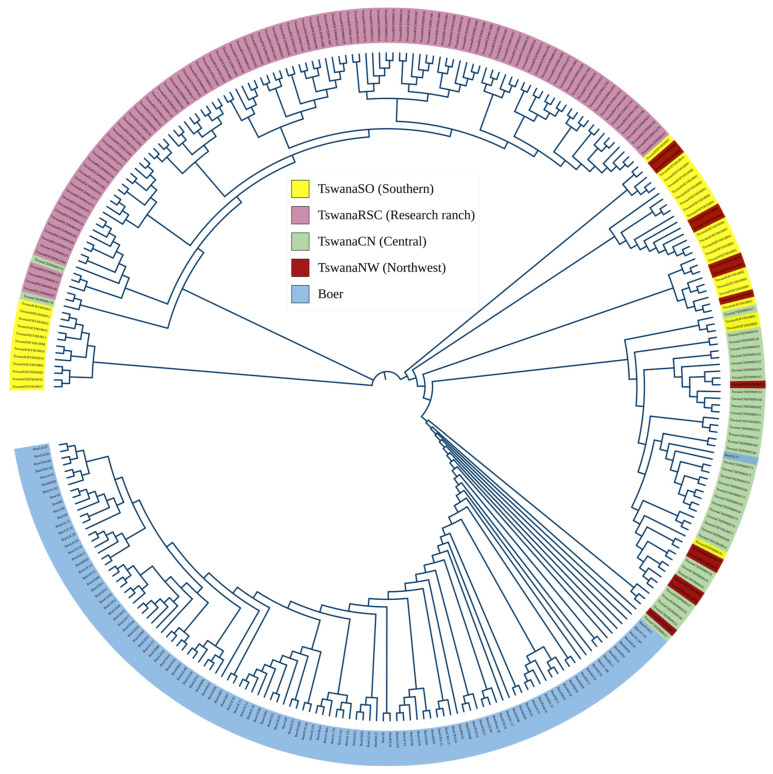
A neighbor joining tree of the Tswana and Boer goat. The tree was constructed using distance base matric of 48,375 SNPs.

The neighbor-joining (NJ) tree (Figure 5) illustrates the genetic relationships among Tswana goat populations from different regions and among Boer goats. Boer goats form a distinct cluster, confirming their genetic divergence from the Tswana populations. Among Tswana goats, individuals from the research ranches (TswanaRSC) exhibit tight clustering, while TswanaSO (Southern) and TswanaCN (Central) display moderate dispersion, suggesting higher genetic variation. TswanaNW (Northwest) individuals show some admixture with other Tswana populations. These patterns indicate population structuring influenced by geographic distribution and breeding practices.

## 4. Discussion

Within the diverse landscape of livestock genetics, the Tswana goat population stands out as an intriguing focus, molded over generations by a blend of natural forces and human interventions. Leveraging the high-throughput capabilities of the Illumina Goat_IGGC_65K_v2 BeadChip, our study aimed to identify unique patterns of genetic variation within goats from the three agroecological regions in Botswana.

The proportion of informative SNPs (82%) identified within the Tswana goat breed exhibits a resemblance to the findings reported in prior studies, notably aligning closely with the results elucidated by [14,15,16,17]. However, variations in the SNP call rate thresholds across these studies also have an influence on the proportion of useful SNPs identified, and a lack of standardization may contribute to the differences [15]. The findings in this study indicate that the Illumina Goat_IGGC_65K_v2 BeadChip is a useful genomic tool for studies on indigenous goat populations.

The phylogenetic analysis reinforces the observed genetic patterns, revealing clear clustering between the Tswana and Boer goats, with Boer goats forming a distinct and well-supported clade. This confirms their genetic differentiation, likely due to distinct selection pressures and breeding histories.

Within Tswana goats, the population structure aligns with agro-ecological regions, suggesting potential genetic adaptation to local environments. The Southern and Central populations exhibit higher genetic variation and share a degree of genetic exchange, as indicated by their intermixed but distinct clustering in the phylogenetic tree. This pattern, along with the presence of rare alleles, suggests that while they may be genetically structured, they do not appear to be fully distinct ecotypes but rather interconnected subpopulations influenced by geography and management practices.

In contrast, the Northwest population exhibits lower heterozygosity but a negative inbreeding coefficient (F_IS_ = −0.016 ± 0.064), suggesting an outbreeding tendency. Despite movement restrictions due to Foot and Mouth Disease (FMD) control, new genetic material has entered the region, likely through managed introductions. The phylogenetic tree supports this, showing a more dispersed clustering pattern among Northwest individuals, indicating gene flow from other Tswana subpopulations and even Boer goats, likely due to introgression. However, the small sample size (*n* = 12) limits the strength of this conclusion, necessitating further validation.

The research ranch population (TswanaRSC) displays high heterozygosity, low inbreeding, and moderate minor allele frequencies, reflecting controlled breeding and systematic record-keeping. Its phylogenetic clustering is more cohesive but still shows signs of admixture, consistent with the fact that ranch animals are sourced from communal areas. While this population is managed separately, its genetic profile does not suggest the emergence of a distinct ecotype but rather a well-maintained genetic pool with reduced inbreeding. Similarly, studies on South African goats have shown that breeding practices and animal movement play a greater role in shaping genetic structure than geographical barriers [17].

Generally, our investigation has unveiled elevated levels of expected heterozygosity (*He*) within the Tswana goat population when contrasted with the Boer goat population. The higher effective population size and the linkage disequilibrium findings in the Tswana goat also suggest high genetic diversity. Concurrently, parallels can be drawn with analogous studies on indigenous goat breeds, such as those from South Africa [15,17], Ethiopia [18], Sudan [19], and Cameroon [18], which consistently highlight the trend towards heightened heterozygosity. In the current study, the levels of inbreeding being lowest among Tswana goats (0.077 ± 0.045) and elevated within the commercial Boer breed (0.134 ± 0.054) highlight the differences in allelic diversity between commercial breeds and the often undeveloped indigenous breeds. Similar findings were noted by [20] while investigating breeds under intense selection and those without specific breeding objectives.

However, it is crucial to acknowledge the influence of misattribution, as pointed out by [19], particularly stemming from handlers/farmers misclassifying animals. This discrepancy is evident in individuals labelled as Boer but clustering with the Tswana breed as seen in the population structure analysis. Alternatively, this finding may suggest potential genetic similarities or historical relationships between selected individuals from the Boer goat population and a subgroup within the Tswana goat population.

A decrease in the effective population size over the years is noted for both breeds as in other studies [21,22,23]. The higher effective population size in the Tswana goat, which exceeds *Ne* = 100, suggests that the population is highly diverse and therefore has a greater likelihood of success in improvement and conservation programs [24,25]. The Southern and Central populations exhibit the highest *Ne* values, exceeding 900 in recent generations, and correspondingly low LD levels (average r^2^ ≈ 0.08), suggesting larger breeding populations and limited recent inbreeding or genetic drift. Recent statistics suggest that >700,000 goats are found in the Southern and Central regions, which could contribute to the high effective population sizes (*Ne*) observed in these regions [2]. The larger population base may enhance genetic variability and buffer against inbreeding, thereby sustaining low levels of LD.

In contrast, the Boer population shows the lowest Ne estimates (*Ne* = 240 at generation 53) and highest LD (average r^2^ = 0.16), indicative of a smaller, more intensively selected population with reduced genetic diversity. The Northwest and research ranch groups display intermediate patterns, reflecting moderate *Ne* values (~319–585) and LD levels (r^2^ ≈ 0.11–0.13), possibly due to localized gene flow or historical admixture. These differences likely stem from varying management practices, with indigenous populations (e.g., Southern, Central) maintaining broader genetic bases through communal breeding, while commercial such as the Boer goat or research ranch herds experience stronger artificial selection and genetic bottlenecks.

Despite the valuable insights offered by this study, some limitations should be acknowledged. The relatively small sample sizes from some regions, especially the Northwest (*n* = 12), may limit the power to detect fine-scale genetic structure. Additionally, no samples were obtained from the Ghanzi region due to the absence of phenotypically identified Tswana goats, creating a geographic data gap. Future studies should aim for broader sampling across all agroecological regions, including remote or underrepresented areas, to capture a more comprehensive picture of genetic diversity and possible ecotype differentiation. Furthermore, while this study focused on neutral genetic variation, integrating adaptive genetic markers and whole-genome sequencing data could reveal functional variations linked to local adaptation, disease resistance, or productivity traits. Comparative studies involving other Southern African indigenous and commercial goat breeds would also enhance our understanding of regional genetic relationships and support transboundary conservation efforts.

The Tswana goat maintains substantial genetic diversity, which should be preserved through structured breeding efforts. Promoting community-based breeding programs that incorporate pedigree recording, buck rotation, and strategic sire selection can help avoid inbreeding and maintain genetic health. Training and capacity building for farmers on basic genetic principles, along with increased access to genotyping and advisory support, would empower communities to improve productivity while conserving this important indigenous resource. Policymakers and extension officers are encouraged to support the development of locally appropriate breeding strategies and to integrate genomic tools into livestock development plans.

## 5. Conclusions

This study provides the first comprehensive genomic assessment of the Tswana goat across multiple agroecological regions in Botswana. The results demonstrate that Tswana goats possess high genetic diversity, reflected by elevated heterozygosity, higher minor allele frequency, and a larger effective population size (*Ne*) compared to the intensively selected Boer goat breed. The regional substructure was evident, with Southern and Central populations exhibiting greater diversity and larger *Ne* values, likely due to extensive communal management systems and larger population sizes. In contrast, the Northwest and research ranch populations showed moderate diversity, influenced by localized breeding and possible introgression. Despite these regional differences, no distinct ecotypes were observed, suggesting that Tswana goats form a genetically interconnected population shaped by both environmental and human factors. The lower levels of inbreeding and faster linkage disequilibrium decay support the idea of broad genetic mixing, especially in communal settings. These findings underscore the importance of preserving the genetic integrity of the Tswana goat through structured, regionally tailored breeding programs. Stakeholders should prioritize community-based breeding approaches that incorporate sire rotation, pedigree recording, and farmer education. Such efforts will enhance productivity, support adaptation to diverse environments, and safeguard this valuable genetic resource for future generations.

## Figures and Tables

**Table 1:** Quality control summary of SNP data for Tswana and Boer goats.

Breed	*n*	Removed Animals	Excluded SNPs				
			MAF < 5%	SNP (Call Rate < 95%)	HWE (0.00001)	Total SNPs Removed	Total SNPs Retained
Tswana	192	2	1538	8684	76	10,298	47,037
Boer	112	1	2967	62,618	92	65,677	45,627
Total	304	3					

*n*: sample size, MAF: minor allele frequency, SNP: single nucleotide polymorphism, and HWE: Hardy–Weinberg equilibrium.

**Table 2:** Estimates of genetic diversity indices in the Tswana goat population from the three agroecological regions and research ranches.

Group	Region/Origin	*n*	*Ho* ± s.d.	*He* ± s.d.	MAF ± s.d.	F_IS_ ± s.d.
TswanaCN	Central	39	0.394 ± 0.000	0.401 ± 0.000	0.320 ± 0.122	0.019 ± 0.051
TswanaSO	Southern	32	0.397 ± 0.000	0.399 ± 0.000	0.318 ± 0.123	0.005 ± 0.038
TswanaNW	Northwest	12	0.364 ± 0.000	0.358 ± 0.000	0.289 ± 0.132	−0.016 ± 0.064
TswanaRSC	Ranches	107	0.394 ± 0.000	0.380 ± 0.000	0.300 ± 0.132	−0.038 ± 0.042

*n*: sample size, *Ho*: observed heterozygosity, *He*: expected heterozygosity, MAF: minor allele frequency, F_IS_: inbreeding coefficient, and s.d: standard deviation.

**Table 3:** Estimates of genetic diversity indices in Tswana and Boer goat.

Breed	*Ho* ± s.d.	*He* ± s.d	MAF ± s.d	F_IS_ ± s.d
Tswana	0.395 ± 0.019	0.400 ± 0.000	0.313 ± 0.127	0.013 ± 0.047
Boer	0.367 ± 0.022	0.375 ± 0.000	0.287 ±0.136	0.019 ± 0.060

*Ho*: observed heterozygosity, *He*: expected heterozygosity, MAF: minor allele frequency, F_IS_: inbreeding coefficient, and s.d: standard deviation.

## Data Availability

Data used in this study may be acquired upon request from the corresponding author at pmonau@buan.ac.bw.

## Supplementary Materials

The following supporting information can be downloaded at: https://www.mdpi.com/article/10.3390/genes16060678/s1, Table S1: Comparison of linkage disequilibrium decay between Boer and Tswana goat subpopulations.

## Abbreviations

The following abbreviations are used in this manuscript:

NARDI	National agricultural research and development institute
LD	Linkage disequilibrium
PCA	Principal component analysis
MAF	Minor allele frequency
HWE	Hardy–Weinberg Equilibrium
SNP	Single nucleotide polymorphism
*n*	Number of samples
*Ho*	Observed heterozygosity
*He*	Expected heterozygosity
F_IS_	Inbreeding coefficient
r^2^	Squared correlation coefficient
*Ne*	Effective population size
