# Supplementary material for: Evaluating Genetic Diversity and Regional Variation in Tswana Goats of Botswana
Source: Genes (Basel). 2025 May 30;16(6):678. doi: 10.3390/genes16060678 (PMC12193357; doi:10.3390/genes16060678)
Supplement: Supplementary file 1 [file genes-16-00678-s001.zip › genes-3627345-supplementary.pdf]

Table S1 Comparison of linkage disequilibrium decay between Boer and Tswana goat subpopulations

CHR	Population	Average r^2	Standard Deviation	Average Distance (Mb)
**1**	Boer	0.16	0.18	0.25
**1**	Central	0.08	0.12	0.25
**1**	Northwest	0.13	0.16	0.25
**1**	Research	0.11	0.15	0.25
**1**	Southern	0.08	0.11	0.25
**2**	Boer	0.15	0.17	0.25
**2**	Central	0.07	0.1	0.25
**2**	Northwest	0.13	0.16	0.25
**2**	Research	0.1	0.14	0.25
**2**	Southern	0.08	0.11	0.25
**3**	Boer	0.17	0.19	0.26
**3**	Central	0.08	0.11	0.26
**3**	Northwest	0.13	0.16	0.26
**3**	Research	0.11	0.14	0.26
**3**	Southern	0.07	0.11	0.26
**4**	Boer	0.14	0.16	0.25
**4**	Central	0.08	0.11	0.25
**4**	Northwest	0.13	0.15	0.25
**4**	Research	0.11	0.15	0.25
**4**	Southern	0.08	0.11	0.25
**5**	Boer	0.15	0.17	0.26
**5**	Central	0.08	0.11	0.26
**5**	Northwest	0.13	0.16	0.26
**5**	Research	0.11	0.14	0.26
**5**	Southern	0.08	0.11	0.26
**6**	Boer	0.2	0.21	0.25
**6**	Central	0.09	0.13	0.25
**6**	Northwest	0.14	0.17	0.25
**6**	Research	0.14	0.18	0.25
**6**	Southern	0.09	0.12	0.25
**7**	Boer	0.18	0.2	0.25
**7**	Central	0.08	0.11	0.26
**7**	Northwest	0.13	0.15	0.26
**7**	Research	0.11	0.14	0.26
**7**	Southern	0.08	0.11	0.26

**8**	Boer	0.18	0.2	0.25
**8**	Central	0.08	0.11	0.25
**8**	Northwest	0.13	0.16	0.25
**8**	Research	0.11	0.14	0.25
**8**	Southern	0.08	0.11	0.25
**9**	Boer	0.15	0.17	0.25
**9**	Central	0.08	0.11	0.25
**9**	Northwest	0.12	0.15	0.25
**9**	Research	0.11	0.14	0.25
**9**	Southern	0.08	0.1	0.25
**10**	Boer	0.14	0.16	0.25
**10**	Central	0.07	0.1	0.25
**10**	Northwest	0.12	0.15	0.25
**10**	Research	0.1	0.13	0.25
**10**	Southern	0.07	0.1	0.25
**11**	Boer	0.16	0.18	0.25
**11**	Central	0.08	0.11	0.25
**11**	Northwest	0.12	0.15	0.25
**11**	Research	0.11	0.15	0.25
**11**	Southern	0.08	0.11	0.25
**12**	Boer	0.18	0.19	0.25
**12**	Central	0.08	0.12	0.25
**12**	Northwest	0.13	0.16	0.25
**12**	Research	0.11	0.15	0.25
**12**	Southern	0.08	0.12	0.25
**13**	Boer	0.17	0.19	0.26
**13**	Central	0.07	0.11	0.26
**13**	Northwest	0.12	0.15	0.26
**13**	Research	0.1	0.14	0.26
**13**	Southern	0.07	0.1	0.26
**14**	Boer	0.18	0.2	0.25
**14**	Central	0.08	0.11	0.26
**14**	Northwest	0.13	0.16	0.25
**14**	Research	0.1	0.13	0.26
**14**	Southern	0.07	0.11	0.25
**15**	Boer	0.16	0.18	0.25
**15**	Central	0.07	0.09	0.25
**15**	Northwest	0.11	0.14	0.25
**15**	Research	0.1	0.13	0.25
**15**	Southern	0.07	0.09	0.25
**16**	Boer	0.13	0.16	0.25

**16**	Central	0.08	0.11	0.25
**16**	Northwest	0.12	0.15	0.25
**16**	Research	0.11	0.14	0.25
**16**	Southern	0.08	0.11	0.25
**17**	Boer	0.17	0.19	0.25
**17**	Central	0.09	0.12	0.26
**17**	Northwest	0.13	0.16	0.26
**17**	Research	0.11	0.14	0.26
**17**	Southern	0.07	0.11	0.26
**18**	Boer	0.15	0.19	0.26
**18**	Central	0.09	0.13	0.26
**18**	Northwest	0.14	0.17	0.26
**18**	Research	0.11	0.15	0.26
**18**	Southern	0.09	0.13	0.26
**19**	Boer	0.16	0.18	0.26
**19**	Central	0.07	0.11	0.26
**19**	Northwest	0.12	0.15	0.26
**19**	Research	0.1	0.13	0.26
**19**	Southern	0.07	0.1	0.26
**20**	Boer	0.17	0.18	0.25
**20**	Central	0.09	0.12	0.25
**20**	Northwest	0.14	0.16	0.25
**20**	Research	0.11	0.14	0.25
**20**	Southern	0.08	0.11	0.25
**21**	Boer	0.13	0.16	0.24
**21**	Central	0.07	0.1	0.25
**21**	Northwest	0.12	0.15	0.25
**21**	Research	0.11	0.14	0.25
**21**	Southern	0.07	0.1	0.25
**22**	Boer	0.16	0.19	0.26
**22**	Central	0.08	0.11	0.26
**22**	Northwest	0.12	0.15	0.26
**22**	Research	0.11	0.15	0.26
**22**	Southern	0.08	0.11	0.26
**23**	Boer	0.13	0.16	0.25
**23**	Central	0.06	0.09	0.25
**23**	Northwest	0.11	0.14	0.25
**23**	Research	0.1	0.13	0.25
**23**	Southern	0.06	0.09	0.25
**24**	Boer	0.17	0.18	0.24
**24**	Central	0.08	0.11	0.24

**24**	Northwest	0.12	0.15	0.24
**24**	Research	0.11	0.14	0.24
**24**	Southern	0.07	0.1	0.24
**25**	Boer	0.18	0.19	0.25
**25**	Central	0.07	0.11	0.25
**25**	Northwest	0.12	0.15	0.25
**25**	Research	0.11	0.14	0.25
**25**	Southern	0.08	0.11	0.25
**26**	Boer	0.14	0.17	0.25
**26**	Central	0.07	0.11	0.25
**26**	Northwest	0.11	0.14	0.25
**26**	Research	0.11	0.14	0.25
**26**	Southern	0.07	0.1	0.25
**27**	Boer	0.14	0.16	0.25
**27**	Central	0.09	0.12	0.25
**27**	Northwest	0.12	0.15	0.25
**27**	Research	0.12	0.16	0.25
**27**	Southern	0.07	0.11	0.25
**28**	Boer	0.14	0.16	0.24
**28**	Central	0.07	0.1	0.25
**28**	Northwest	0.12	0.14	0.25
**28**	Research	0.11	0.15	0.25
**28**	Southern	0.07	0.1	0.25
**29**	Boer	0.15	0.17	0.26
**29**	Central	0.07	0.1	0.27
**29**	Northwest	0.12	0.14	0.27
**29**	Research	0.09	0.12	0.27
**29**	Southern	0.07	0.09	0.27
